# Emerging wearable technologies for multisystem monitoring and treatment of Parkinson’s disease: a narrative review

**DOI:** 10.3389/fnetp.2024.1354211

**Published:** 2024-02-13

**Authors:** Yasmine M. Kehnemouyi, Todd P. Coleman, Peter A. Tass

**Affiliations:** ^1^ Department of Neurosurgery, Stanford University School of Medicine, Stanford, CA, United States; ^2^ Department of Bioengineering, Stanford University School of Engineering, Stanford, CA, United States

**Keywords:** Parkinson’s disease, wearable technologies, motor, cognition, gastrointestinal, autonomic, sleep, monitoring

## Abstract

Parkinson’s disease (PD) is a chronic movement disorder characterized by a variety of motor and nonmotor comorbidities, including cognitive impairment, gastrointestinal (GI) dysfunction, and autonomic/sleep disturbances. Symptoms typically fluctuate with different settings and environmental factors and thus need to be consistently monitored. Current methods, however, rely on infrequent rating scales performed in clinic. The advent of wearable technologies presents a new avenue to track objective measures of PD comorbidities longitudinally and more frequently. This narrative review discusses and proposes emerging wearable technologies that can monitor manifestations of motor, cognitive, GI, and autonomic/sleep comorbidities throughout the daily lives of PD individuals. This can provide more wholistic insight into real-time physiological *versus* pathological function with the potential to better assess treatments during clinical trials and allow physicians to optimize treatment regimens. Additionally, this narrative review briefly examines novel applications of wearables as therapy for PD patients.

## 1 Introduction

Parkinson’s disease (PD) is the second most common neurodegenerative disease and most common movement disorder. The incidence of PD in the U.S. is approximately 20 cases per 1,00,000 people per year, and its prevalence is reported to be approximately 1% in people 60 years of age and older (DeMaagd and Philip, 2015). PD is difficult to diagnose and treat early, as it shows wide variability in clinical presentation (Rovini et al., 2017). The most common clinical manifestation of PD is motor dysfunction, including bradykinesia, freezing of gait (FOG), and tremor. Standard of care PD diagnosis is based on the severity of these motor symptoms, quantified in the clinic by a neurologist using the Unified Parkinson’s disease Rating Scale (MDS-UPDRS III) (Hendricks and Khasawneh, 2021).

While aspects of motor dysfunction may act as disease progression markers, there is growing evidence of a prodromal, nonmotor phase that may begin decades before motor symptoms are present. Comorbidities such as cognitive, gastrointestinal (GI), and autonomic/sleep dysfunction are hard to manage and significantly affect the quality of life of PD individuals (Del Tredici and Braak, 2016). Monitoring is inconsistently performed through rating scales (Goldman and Postuma, 2014). This introduces subjectivity via clinician interpretation and a lack of fine-grained specificity in symptom characterization. Additionally, measuring this wide array of comorbidities in the clinic becomes difficult when symptoms manifest transiently and do not always appear in front of a neurologist. Wholistic disease management thus creates an opportunity to view PD as a multisystem disorder which includes assessment and treatment of both motor and nonmotor symptoms. Objective measures of symptoms continuously monitored in and outside the clinic are necessary to understand the disease course beyond what is possible in a clinic visit’s time constraints, with the potential to enable prompt, more personalized therapeutic interventions for PD management.

In recent years, wearable technologies have emerged as promising tools to characterize PD symptoms. As most recently noted, wearables track symptom progression better than conventionally used rating scales and present a portable, non-invasive means to monitor many comorbidities in real-time. One can also gain more mechanistic insight into the pathology of motor and nonmotor systems in PD by analyzing spatiotemporal features tracked by wearables (Sotirakis et al., 2023). This is extremely crucial for improving assessments during clinical trials and to optimize treatment regimens with much less time needed in the clinic. Thus, this review will discuss and propose the use of emerging wearable technologies for monitoring physiological manifestations of four main systems that go awry in PD, namely the motor, cognitive, GI, and autonomic/sleep networks. It will also discuss the advent of wearable technologies with actuators as novel interventions, more specifically the use of wearables to deliver personalized therapy.

## 2 Motor system

### 2.1 Physiological manifestation of motor dysfunction in PD

Common motor deficits in PD include loss of voluntary movement (akinesia) and the slowing of movements (bradykinesia). People with PD thus have difficulty executing repetitive movements. Additional comorbidities include gait impairment and tremor (Moustafa et al., 2016). Cardinal motor signs manifest in PD due to a reduction of dopamine levels in the basal ganglia circuitry (substantia nigra) due to aggregation of misfolded alpha-synuclein proteins, termed Lewy bodies, which propagate throughout the central nervous system (CNS) (Gómez-Benito et al., 2020). Computational models suggest that the source of bradykinesia (and eventually akinesia) involves over-excitability of striatal neurons, with inhibitory projections to cortical areas (McGregor and Nelson, 2019). Basal ganglia neuropathology also contributes to gait impairment and freezing of gait (FOG), resulting in increased gait variability and reduced range of motion (Hausdorff, 2009). Bradykinesia and gait impairment typically manifest as progressively reduced amplitude and frequency throughout bouts of movement. Additionally, PD rest tremor has been associated with dysfunction in the cerebellum, thalamus, and subthalamic nucleus (STN), specifically due to abnormal synchrony between the basal ganglia-thalamocortical and cerebellar-thalamocortical circuits. Tremor manifests as asymmetric movement with medium amplitude at typical frequencies of 4–9 Hz (Lefaivre et al., 2016).

### 2.2 Emerging technologies in assessing motor function

Wearable sensors have emerged as valuable tools to monitor motor symptom progression longitudinally (Sotirakis et al., 2023). These technologies can capture personalized, continuous kinematics both in and outside the clinic. Typically, inertial measurement units (IMUs) are placed on various limbs of subjects, including the feet, shanks, knees, etc., and their built-in accelerometers, gyroscopes, and magnetometers can record different features of PD movement. A recent study using IMUs coupled with convolutional neural network modeling to track FOG in PD subjects explored sensor locations that maximized patient adherence for personalized at-home motor symptom monitoring (O Day et al., 2022). Additionally, smartphones can measure spatiotemporal features of rest tremor using a three-dimensional built-in accelerometer and robust software packages. For instance, a 2010 study used an iPhone strapped to the dorsum of the hand to explore spectral features of PD tremor and found dominant frequencies between 5 and 10 Hz, while minimal frequency content was found in the healthy control cohort, suggesting that smartphone accelerometers are reliable for PD tremor longitudinal monitoring (Lemoyne et al., 2010).

## 3 Nonmotor system

### 3.1 Cognition

#### 3.1.1 Physiological manifestation of cognitive dysfunction in PD

Cognitive impairment, a PD symptom that is extremely heterogenous in manifestation throughout the disease course, may initially be attributed to loss of 30%–70% of cholinergic neurons in the nucleus basalis of Meynert (NBM). Initial studies have shown that in PD individuals with dementia, early alpha-synuclein inclusions have been found in the NBM (Wilson et al., 2021). Additionally, a severe loss of cholinergic neurons in the basal forebrain has been shown to differentiate PD individuals with and without dementia. Thus, it has been hypothesized that alpha-synuclein deposition in basal forebrain nuclei drives terminal cholinergic dysfunction and leads to the development of PD dementia (Pasquini et al., 2021). Finally, reductions in cholinergic signaling have been found in parieto-occipital regions that then spread to frontal and temporal cortices in cognitively impaired individuals with PD (Bohnen et al., 2018). This loss of cortical and subcortical cholinergic neurotransmission is associated with impairments in attention, executive function, visual perception, and memory (Pasquini et al., 2021; van der Zee et al., 2021).

Using electroencephalography (EEG), event related potentials (ERPs) can be used to quantitatively assess cognitive state. ERPs are averaged EEG activity that is time locked to a repetitive sensory stimulus. Typically, ERP latencies are related to the time course of cognitive processes, such as the evaluation of a stimulus and preparation of a response. ERP amplitudes indicate the extent to which neural resources are recruited for these processes. Features of impaired PD cognition include a large ERP amplitude at 300 ms post-stimulus (known as P300), indicative of impaired behavioral responses to auditory, visual, or somatosensory stimuli. Cognitive state has been associated with power spectral density features such as increased alpha power related to dysfunctional memory load (Seer et al., 2016).

#### 3.1.2 Emerging technologies in assessing cognitive function

The development of wearable technologies to assess cognitive impairment presents an emerging opportunity to track subtleties in cognitive decline. This allows for more frequent but targeted cognitive batteries and neuropsychiatric tests that do not require extensive time and money at a clinical visit. Smartwatch and smartphone-based cognitive assessments serve as a tool to measure cognition in real-time outside of the clinic environment, as cognition may fluctuate with exercise, sleep, stress, and social settings (Hays et al., 2019). A 12-month longitudinal study explored monitoring of cognitive and psychomotor functioning in PD via smartphone app, *BrainBaseline* (Adams et al., 2023). PD subjects and healthy controls performed cognitive batteries such as Trail Making Test Part A, assessing complex attention, and Symbol Digit Modalities Test, assessing psychomotor speed. Of note, lower performance on the standard cognitive battery correlated with worsened performance on these smartphone cognitive assessments. Along with the ability to synchronously collect other digital biomarkers such as step information, these results are promising to frequently monitor cognitive and psychomotor functioning (under cognitive load) throughout one’s daily life (Crétot-Richert et al., 2023).

Recent work has used in-ear EEG to explore spatiotemporal features related to focus, specifically working memory and cognitive function under load. In-ear EEG refers to recording of EEG activity from electrodes placed near or in the ear. Compared to conventional scalp EEG, in ear-EEG is more comfortable, customizable, portable, and avoids inconsistencies with optimizing impedances between the electrode-skin interface (Crétot-Richert et al., 2023). Previous studies were first to demonstrate its capability to record reliable ERPs and spectral features related to aspects of cognition such as attention. They too detected robust spectral features indicative of cognitive workload and working memory (Mirkovic et al., 2016).

### 3.2 Gastrointestinal

#### 3.2.1 Physiological manifestation of gastrointestinal dysfunction in PD

GI dysfunction in PD may result from pathological alpha synuclein aggregates in the enteric nervous system (ENS) and brainstem areas such as the dorsal motor vagal nucleus (DMV) and nucleus tractus solitarius (NTS) (Fasano et al., 2015; Bove and Travagli, 2019). Enteric and brainstem circuitry play a vital role in vagal regulation of peripheral metabolic functions. Afferent and efferent vagal fibers regulate metabolic homeostasis by modulating visceral functions, such as GI motility. Additionally, the vagus nerve is a major constituent of the inflammatory reflex, whose activation results in modulating digestive function (Pavlov and Tracey, 2012). Thus, vagal denervation plays an important role in PD GI dysfunction, manifesting in symptoms throughout the entire GI tract, i.e. swallowing and esophageal motility abnormalities, gastroparesis, and constipation (Kim and Sung, 2015). 70%–100% of PD individuals experience gastric motility issues, and roughly half have reported symptoms of gastroparesis in early and late disease stages (Soliman et al., 2021). Nausea, vomiting, early satiety, excessive fullness, bloating, and abdominal distension characterize gastroparesis, many often left untreated and increase in severity over time (Fasano et al., 2015; Kim and Sung, 2015).

#### 3.2.2 Emerging technologies in assessing gastrointestinal function

The multifaceted role of the GI system in PD calls for the development of wearable technologies to objectively, non-invasively, and more sensitively assess physiology in concert with symptom reporting to help develop personalized treatment plans. A recent 2021 finding noninvasively assessed irregular gastric myoelectrical activity using a four-channel electrogastrogram (EGG) placed on the surface of the abdomen in early-stage PD subjects compared against healthy controls. They explored objective spatiotemporal metrics such as gastric slow wave frequency and EGG power, preprandially and postprandially, and found irregular gastric slow wave activity in the PD patients preprandially, likely alluding to gastric dysmotility in PD (Araki et al., 2021). To gain insight into mechanisms of specific GI symptoms and potential treatments in PD, it is important to be able to tease apart symptoms and attribute them to detailed diagnoses. The development of the high-resolution EGG (HR-EGG) enables the tracking of spatial slow-wave abnormalities extracted from cutaneious multi-electrode recordings (Gharibans et al., 2018). The amount of irregularity in spatial slow wave patterns extracted from HR-EGG cutaneously has also been shown correlate with symptom severity in patients with functional dyspepsia and gastroparesis (Gharibans et al., 2019). Modern applied probability approaches using robust regression and optimal transport theory were also recently used in concert with HR-EGG and symptom reports to determine when gastric slow wave (as compared to other etiologies) gives rise to symptoms in patients with functional dyspepsia and gastroparesis (Agrusa et al., 2022). Roughly half of PD patients have gastroparesis; constipation, colonic dysmotility, and vagal atrophy are also quite common co-occurrences (Walter et al., 2018). As such, these tools, which have recently shown to be deployable in an ambulatory setting, could be explored to determine in what contexts spatial gastric slow wave abnormalities as compared to other etiologies give rise to symptoms in PD individuals (Gharibans et al., 2018; Subramanian et al., 2023).

The advent of ingestible devices has brought upon novel ways to monitor gastric motility and diagnose GI disorders. Traverso et al. developed a small ingestible bioelectronic sensor that can diagnose GI motility disorders that are otherwise invasive to specifically diagnose and monitor (Sharma et al., 2023). Upon ingestion, the sensor adheres to the stomach wall or intestinal lining and can measure rhythmic contractions of the GI tract for multiple days. In connection with a smartphone, the ingestible device can take consistent, convenient, non-invasive internal recordings of the GI tract. This has the potential to provide PD individuals with valuable insight into the state of their GI motility and provides means for rapid, earlier clinical evaluation and intervention as necessary (Sharma et al., 2023).

### 3.3 Autonomic/sleep

#### 3.3.1 Physiological manifestation of autonomic/sleep dysfunction in PD

ANS dysfunction in PD may attribute to alpha synuclein aggregates and autonomic nerve denervation in peripheral sympathetic, parasympathetic, and enteric nervous systems prior to manifestation of central neuropathology. Central autonomic control centers that experience Lewy pathology include cortical regions such as the insula, hypothalamus, and brainstem regions (NTS, DMV) (Chen et al., 2020). Symptoms of dysautonomia include cardiovascular dysregulation (orthostatic hypotension (OH), heart rate variability (HRV)) and sleep dysfunction; these manifest at varying rates of occurrence and intensities throughout the disease (Palma and Kaufmann, 2018; Chen et al., 2020). OH in PD (∼32% prevalence) refers to a failure of the ANS to regulate blood pressure in response to postural changes due to inadequate release of norepinephrine (Chen et al., 2020; Dommershuijsen et al., 2021). This may result from abnormal response of the cardiovascular regulatory center to a reduction of blood pressure from a body position change (Dommershuijsen et al., 2021). Furthermore, PD individuals that experience cardiac denervation may also have cardiovascular dysfunction, or destruction of the sympathetic and/or parasympathetic nervous systems that contribute to imbalances observed in blood pressure (BP) and HRV (Chen et al., 2020). This affects roughly 80% of PD patients and worsens with disease progression. Typically parasympathetic activity is decreased while sympathetic activity is increased, and vagus atrophy is associated with impaired heart rate variability in PD (Chen et al., 2020; Cuenca-Bermejo et al., 2021).

Many people with PD also prodromally experience sleep dysfunction, specifically idiopathic REM sleep behavior disorder (iRBD) (Postuma et al., 2015). This manifests as early Lewy pathology in brainstem areas resulting in a loss of atonia during REM sleep (Boucetta et al., 2016). iRBD is an extremely accurate predictor of Lewy body disorder: roughly 90% of iRBD patients develop PD or Lewy body dementia. Evidence shows a close association between iRBD and dysautonomia, i.e. people with PD-iRBD experience low HRV (Riboldi et al., 2022).

#### 3.3.2 Emerging technologies in assessing autonomic/sleep function

Monitoring these features of PD dysautonomia are crucial for tracking disease progression but have proven quite difficult thus far. Autonomic comorbidities manifest variably throughout the day based on activity and circadian patterns as well as throughout the disease course (Videnovic and Golombek, 2013). Regular measurements of BP in PD individuals are crucial to detect irregular fluctuations, inform disease management, and reduce cardiovascular and fall risks (Ahn et al., 2021; Tulbă et al., 2021). As such, technologies such as cuff-less BP monitors in the form of a wristwatch have come to fruition. In 2021, Ahn et al. were the first to validate the use of a smartwatch to monitor BP using photoplethysmography (PGG) in PD subjects. They found that measuring BP using PPG was a reliable monitoring modality in PD regardless of time of day or environment (home/clinic). This can foster real-time monitoring of autonomic state for more timely PD diagnosis and proper management (Ahn et al., 2021). Additionally, the statistical nature of timings of heartbeats has proven a robust measure of autonomic tone; it is important for PD management to promptly capture subtleties of heartbeat fluctuations (Siepmann et al., 2022). A recent finding studied the statistics of heart interbeat intervals in diabetic gastroparesis patients by instrumenting them wearable ECG monitoring device. They extracted heartbeat dynamics from ECG signals and sleep duration from a built-in accelerometer to capture dynamic measures of sympathetic and parasympathetic balance. They observed the strength of the ratio between low frequency (sympathetic) and high frequency (parasympathetic) activity (LF:HF), specifically during sleep *versus* wake and found that measures of reduced autonomic innervation throughout the day as well as a reduction of parasympathetic innervation during sleep as compared to wakefulness in the diabetic dysautonomic subjects (Subramanian et al., 2023). Non-invasive monitoring using this paradigm can be applicable to other conditions with dysautonomia such as PD (Hellman et al., 2015). As ingestible devices have been used for measuring gastric motility, they have also recently been developed to monitor breathing rate and heart rate. Rather than spending many hours being monitored in clinic or laboratories, such ingestible devices to measure a patient’s breathing rate and heart rate overnight using an embedded accelerometer, may be useful for a variety of diagnoses involving irregular autonomic function and respiratory function (Traverso et al., 2023).

### 3.4 Discussion

**FIGURE 1:**
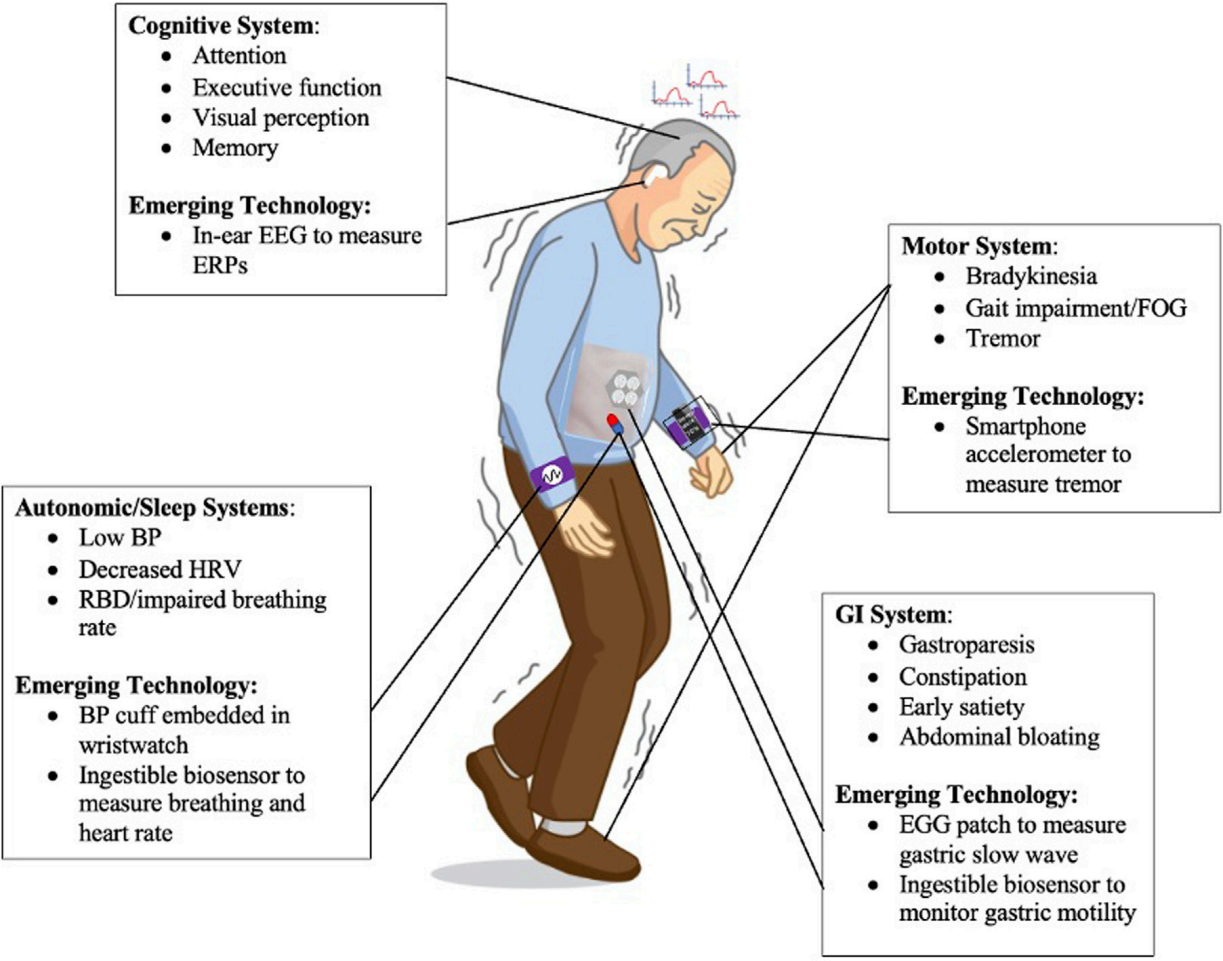
Motor System: IMUs built into a smartphone can monitor personalized kinematics of PD motor symptoms such as tremor. Cognitive System: One can frequently track cognitive and psychomotor functioning by measuring ERPs using in-ear EEG. GI System: It is now possible to assess one’s GI function and diagnose GI conditions using EGG sensors placed on the abdomen. Autonomic/Sleep Systems: Wearable technologies, including a wristwatch embedded with BP monitoring, can measure real-time autonomic state of PD individuals.

Wearable technologies have unique potential to track and monitor objective measures of motor and nonmotor symptoms of PD (Figure 1). Wearable IMUs for instance are valuable tools to monitor personalized kinematics in a variety of settings outside the clinic. Also, one can frequently assess cognitive and psychomotor functioning via smartphone applications and portable physiological sensors. It is now possible to obtain more insight into the state of one’s GI function, including specific GI disorders, with the advent of wearable multi-electrode EGG monitoring techniques and ingestible devices. Finally, wearables are ideal to measure real-time autonomic state, for example using cuff-less BP monitoring and ingestible devices that measure respiratory function. These solutions combat the limitations regarding inconsistencies via insensitive, infrequent rating scales and questionnaires, inter-rater variability, and symptom fluctuations in and out of clinic.

Not only are wearable technologies used to inform necessary therapy, but they also have the potential to deliver therapy both in and outside the clinic (Pfeifer et al., 2021; Yu et al., 2020; Moreau et al., 2023). One such wearable technology includes a non-invasive vibrotactile glove that delivers stimulation to the fingertips of PD individuals via embedded mechanical stimulators, used to alleviate motor symptoms such as tremor and gait (Pfeifer et al., 2021). Additionally, augmented reality devices have potential to provide PD individuals with on-demand activities and visual and auditory cueing to improve motor and nonmotor comorbidities, both with and without cognitive load. For example, a dance intervention (with cueing) delivered in the home via smart eyeglasses known as Google Glass was shown to be effective in improving gait performance, balance, postural instability, and cognitive function in PD individuals (Tunur et al., 2020). Finally, wearable technologies can be used to provide physiology-driven signals to inform personalized PD therapies such as deep brain stimulation (DBS). More specifically, velocity from IMUs on the shanks of PD individuals can be used as a kinematic control variable drive adaptive STN-DBS therapy in real-time to improve gait impairment and FOG (Melbourne et al., 2023). Thus, it is important to utilize emerging wearable technologies for PD motor and nonmotor symptom monitoring and treatment to better understand mechanisms of PD, assess these physiologic fluctuations during clinical trials, and monitor both longitudinal disease progression and wholistic treatment efficacy both in and outside the clinic. As more wearable technologies emerge, it is important that sound clinical validation is performed against both motor and nonmotor standard clinical rating scales. The more these novel clinically validated wearables are used in untapped applications, this will continue to push the limits of clinical characterization of the disease, with motivation to keep improving quality of life of people with PD.
